# Assessment of the association of OCT3/4 with GLUT1 and CD105 in oral squamous cell carcinoma using dual immunohistochemistry

**DOI:** 10.1186/s12903-022-02332-w

**Published:** 2022-07-19

**Authors:** Samira Derakhshan, Nazanin Mahdavi, Neda Kardouni Khoozestani, Bita Nasr Esfahani, Foroozan Heidarian, Sedigheh Rahrotaban, Ali Abdolrahmani

**Affiliations:** 1 Department of Oral and Maxillofacial Pathology, School of Dentistry, Tehran University of Medical Sciences, Tehran, Iran; 2 Cancer Preclinical Imaging Group, Preclinical Core Facility, Tehran University of Medical Sciences, Tehran, Iran; 3 School of Dentistry, Tehran University of Medical Sciences, Tehran, Iran; 4 Tehran University of Medical Sciences, Tehran, Iran; 5 Dental Research Center, Dentistry Research Institute, School of Dentistry, Tehran University of Medical Sciences, North Kargar St, Tehran, Iran

**Keywords:** Angiogenesis, Cancer stem cell, Dual immunohistochemistry, GLUT1, OCT3/4, Squamous cell carcinoma

## Abstract

**Background:**

Oral squamous cell carcinoma (OSCC) is the most common cancer affecting the oral and maxillofacial region. This study aimed to investigate the role of cancer stem cells (CSCs) in angiogenesis and hypoxic response in OSCC.

**Methods:**

This retrospective observational study evaluated 56 cases of OSCC using dual immunohistochemistry. Octamer-binding transcription factor 3/4 (OCT3/4) marker was used to evaluate CSC activity. Glucose transporter 1 (GLUT1) marker was used to evaluate the hypoxic response and angiogenesis, while endoglin (CD105) was used to evaluate the late stage of angiogenesis and blood vessel formation.

**Results:**

Co-expression of OCT3/4 and GLUT1 was noted in 11 of 12 patients with grade III OSCC. However, we did not observe co-expression of these markers in 13 of 22 patients with grade I OSCC. Although we observed a significant correlation between co-expression of GLUT1 and OCT3/4 and tumor grade, there was no significant correlation between co-expression of OCT3/4 and CD105 and different grades of OSCC.

**Conclusions:**

CSCs could play important roles in the initial stages of hypoxic response and angiogenesis. Our result reported that in higher grades of OSCC, GLUT1 as a first response to hypoxic situations might be a result of CSCs. Further studies are required to discover other biomarkers, their roles, and associated pathways of CSCs in OSCC.

## Introduction

Oral squamous cell carcinoma (OSCC) accounts for approximately 90% of all oral malignancies. In addition to its high prevalence, OSCC has a poor five-year survival rate of about 50% [1, 2]. Despite the advancements made in surgical techniques and adjuvant therapeutic modalities, the long-term survival rate of OSCC patients has only shown a modest improvement [3]. Therefore, in addition to the conventional TNM staging and histopathological differentiation, finding accurate and reliable prognostic markers to identify high-risk patients could be promising.

Since the identification of cancer stem cells (CSCs) by Bonnet in 1997 [4], their role in the hierarchical organization of cancer cells has received wide attention. In this process, angiogenesis and cellular heterogeneity are maintained by only this small group of cells that have the ability to self-renew and differentiate. These cells may actually be responsible for resistance to radiation and chemotherapy [5]. Thus, CSCs are recognized as one of the most important factors responsible for the failure of conventional cancer therapy. This statement led to the development of a new paradigm in the treatment of malignant lesions [6]. Octamer-binding transcription factor 3/4 (OCT3/4), also known as POU5F1 and Oct4, is a master factor for CSC functions such as self-renewal and differentiation and may play a critical role in developing resistance to conventional cancer therapy [7]. Alterations in the tumor microenvironment, such as hypoxia, have a significant effect on tumor progression and CSC functions through modification of metabolic pathways and production of hypoxia-inducible factor-1α [6, 8]. Uncontrolled oncogene-induced proliferation in the absence of efficient blood supply results in hypoxia. The tumors react to hypoxia by inducing angiogenesis and metabolic remodeling to enhance their progression [9]. Therefore, angiogenesis is fundamental for the progression, invasion, and metastasis of tumors. Many signaling pathways play essential roles in angiogenesis; therefore, angiogenesis can be detected or inhibited through such pathways [10]. At preliminary stages, glucose transporter-1 (GLUT1) expression increases in response to hypoxia-inducible factor-1α [11]. Also, owing to the role of GLUT1 in tumor progression and metabolism, overexpression of GLUT1 could predict more aggressive behavior and a worse clinical outcome [12]. Endoglin (CD105) is part of the transforming growth factor beta receptor complex, which is up-regulated in proliferating endothelial cells [13]. The lack of sufficient information about the correlation between the expression of CSC markers and angiogenesis in head and neck carcinomas, especially the oral squamous cell carcinoma (OSCC) encouraged us to further scrutinize this correlation in OSCC. In order to investigate the role of CSCs in tumor response to hypoxia, we assessed GLUT1 and CD105 as markers of hypoxic response and angiogenesis in this study.

## Materials and methods

### Samples

A total of 56 formalin-fixed, paraffin-embedded tissue specimens diagnosed with OSCC were selected from the archives of the Oral and Maxillofacial Pathology Department of Dental School of Tehran University of Medical Sciences. The blocks were inspected again to confirm the diagnosis and also for the assessment of histological tumor grade. Specimens with optimal tissue adequacy were selected. Histopathological tumor grading was performed according to the 2017 World Health Organization classification of head and neck tumors based on the degree of resemblance to the normal squamous epithelium and the amount of keratin production; graded as well differentiated (grade I), moderately differentiated (grade II), or poorly differentiated (grade III) [14]. Demographic information of patients and clinico-histopathological features were collected from the records. This cross-sectional study was carried out in two phases and supported by Tehran University of Medical Sciences, School of Dentistry grants No #98-01-69-41495 and #98-02-69-42939, and was approved by the ethics in research committee (registration codes IR.TUMS.DENTISTRY.REC.1398.010 and IR.TUMS.DENTISTRY.REC.1399.164).

### Staining

The specimens were sectioned into 3-µm thick slices. Immunohistochemical (IHC) staining was performed using a dual staining kit which is used for simultaneous evaluation of two or more antigens. The master dual staining kit (MAD-001882QK, Master Diagnostica, Granada, Spain) was used according to the provided instructions to evaluate the expression of GLUT1, CD105, and OCT3/4 markers. Two sections of each block were stained. The first section was stained with CD105 and OCT3/4, and the second section was stained with GLUT1 and OCT3/4. Dual staining requires two types of antibodies from two different animal sources; thus, we used goat polyclonal antibodies to detect OCT3/4 and mouse monoclonal antibodies to detect CD105 and GLUT1 (Table 1).

**Table 1:** Antibodies information used for immunohistochemistry examination

Antibody	Type of antibody	Host animal	Brand	Catalog number	Clone	Dilution
OCT3/4	Polyclonal	Rabbit	ABCAM	ab19857	–	1:100
CD105	Monoclonal	Mouse	Biotechne	MAB1320	209701	1:100
GLUT1	Monoclonal	Mouse	Santa Cruz	sc-377228	(A-4)	1:50

### Evaluation

Each stained block was separately scored by two senior pathologists. Brown staining of the nuclei was regarded as a positive result for OCT3/4. The intensity and percentage of staining were both evaluated for OCT3/4 scoring. The intensity of staining was rated from 0 to 3 (0 = no staining, 1 = mild staining, 2 = moderate staining, 3 = severe staining). Distribution of staining was rated from 0 to 3 (0 = no cell is stained, 1 = less than 10% of cells are stained, 2 = 10 to 50% of cells are stained, and 3 = more than 50% of cells are stained). The total score (0–9) was calculated by multiplying the intensity score and distribution score. Scores 0–2 indicated low, 3–4 indicated moderate, and 6–9 indicated severe expression [15].

Red staining of cell cytoplasm was regarded as a positive result for angiogenesis (CD105). The first step in assessing angiogenesis was to determine the highly stained fields (neovascularization hotspots) at low magnification at the invasive edge. After the selection of four hotspot areas, the CD105 positive cells count was performed at 400× magnification, and the CD105 score was determined as the average count of CD105 positive cells in four areas. The median value was considered as a cut-off point to determine the high and low expression of CD105 [16, 17]. The surrounding connective tissue endothelial cells were used as the internal positive control.

For evaluation of GLUT1 immunoreactivity, both membranous and cytoplasmic staining was considered a positive result. The percentage of positively stained tumoral cells was graded as score 0 (no positive cell), 1 (1–10% positive tumoral cells), 2 (11–50% positive tumoral cells), 3 (50–80% positive tumoral cells), and 4 (81–100% positive tumoral cells). Using the median score (2) as a cut-off, scores 0, 1 and 2 were regarded as low expression and scores 3 and 4 were regarded as high expression of GLUT1 [18].

In concurrent staining of cells for angiogenesis markers and OCT3/4 antibody, red cytoplasmic and brown nuclear staining of the same cell was used as the criterion for co-expression and regarded as a positive result. The co-expression score was determined according to the percentage of tumoral cells with a concurrent positive reaction to both antibodies. Co-expression score was categorized as no co-expression (0% of tumoral cells), low co-expression (1–25% of tumoral cells), and high co-expression (> 25% of tumoral cells).

### Data analysis

The data were analyzed using SPSS 25 (IBM SPSS Statistics for Windows, Armonk, NY, USA). The Kruskal–Wallis test was used to compare the expression of CD105 and OCT3/4 separately in tumoral cells and the co-expression of these markers in different OSCC grades by calculating the mean rank. The fisher's exact test was used to compare the expression of GLUT1, OCT3/4, and co-expression of them in different grades of OSCC patients. Also, the spearman correlation test was performed to assess any possible correlation between tumor grade and CD105, GLUT1, OCT3/4, or co-expression of these markers. P values less than 0.05 were considered statistically significant.

## Results

**Fig. 1:**
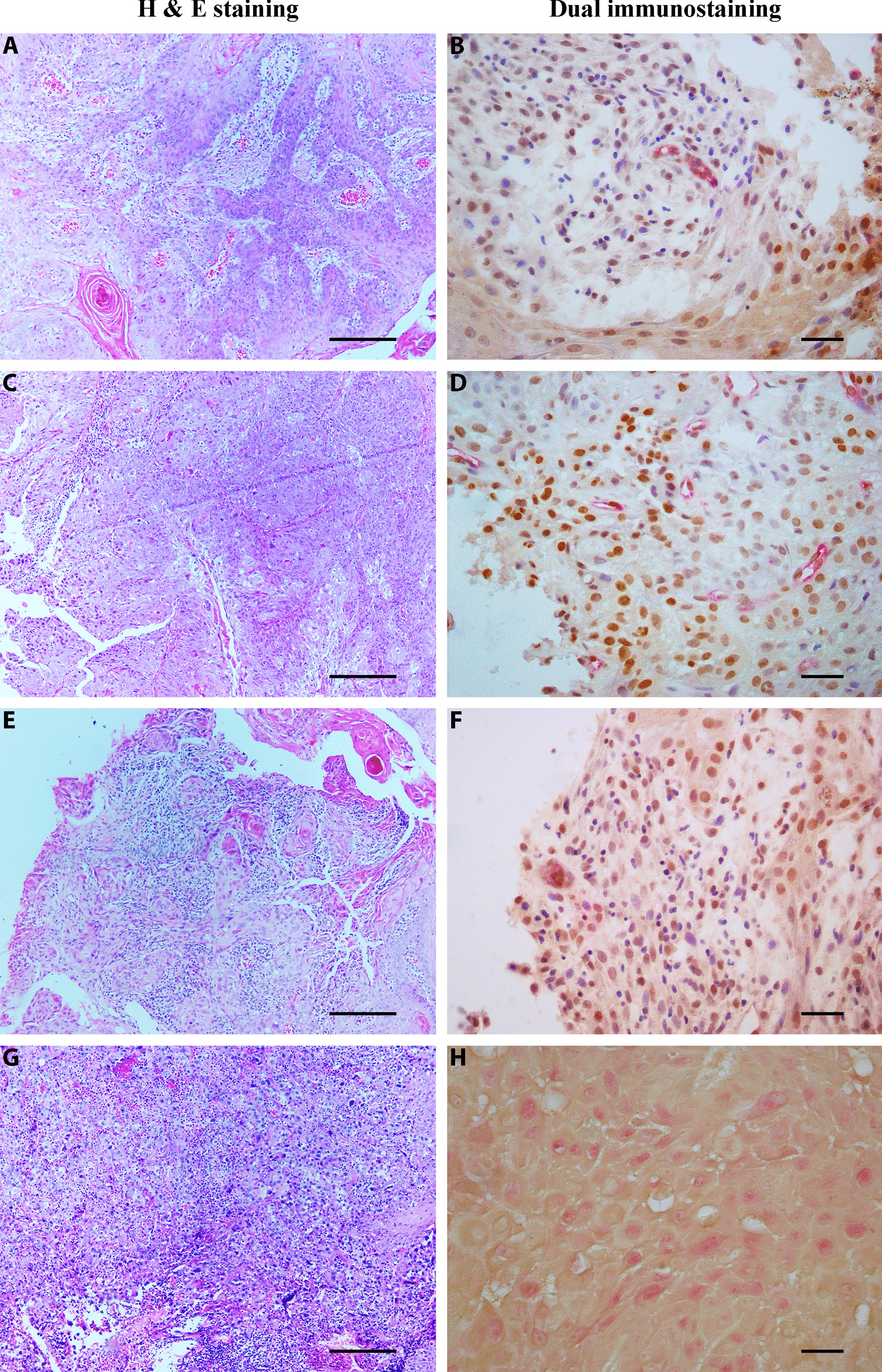
H& E staining and dual immunostaining findings; **A**, **E** Well differentiated SCC (× 100 magnification, 200 μm scale bar). **C**, **G** Poorly differentiated SCC (× 100 magnification, 200 µm scale bar). **B** Low co-expression of CD105 and OCT3/4 in well differentiated SCC; Positive red cytoplasmic immunostaining for CD105 and brown nuclear immunostaining for OCT3/4 in tumoral cells (× 400 magnification, 30 µm scale bar). **D** High co-expression of CD105 and OCT3/4 in poorly differentiated SCC; Positive red cytoplasmic immunostaining for CD105 and brown nuclear immunostaining for OCT3/4 in tumoral cells (× 400 magnification, 30 µm scale bar). **F** Low co-expression of GLUT1 and OCT3/4 in well differentiated SCC; Positive red cytoplasmic immunostaining for GLUT1 and brown nuclear immunostaining for OCT3/4 (× 400 magnification, 30 µm scale bar). **H** High co-expression of GLUT1 and OCT3/4 in poorly differentiated SCC; Positive red cytoplasmic immunostaining for GLUT1 and brown nuclear immunostaining for OCT3/4 (× 400 magnification, 30 µm scale bar)

Positive immunoreactivity of tumoral cells in the dual staining method is shown in Fig. 1. Tumor samples belonged to 21 female (37.5%) and 35 male (62.5%) patients. The histopathological grade was grade I in 27 patients, grade II in 15 cases, and grade III in 14 cases (Table 2).

**Ta﻿ble 2:** Tumor grade and gender distribution

		Gender		Total
		Female	Male	
Grade				
I	Count	9	18	27
	% within grade	33.3%	66.7%	100.0%
II	Count	8	7	15
	% within grade	53.3%	46.7%	100.0%
III	Count	4	10	14
	% within grade	28.6%	71.4%	100.0%
Total	Count	21	35	56
	% within grade	37.5%	62.5%	100.0%

**Fig. 2:**
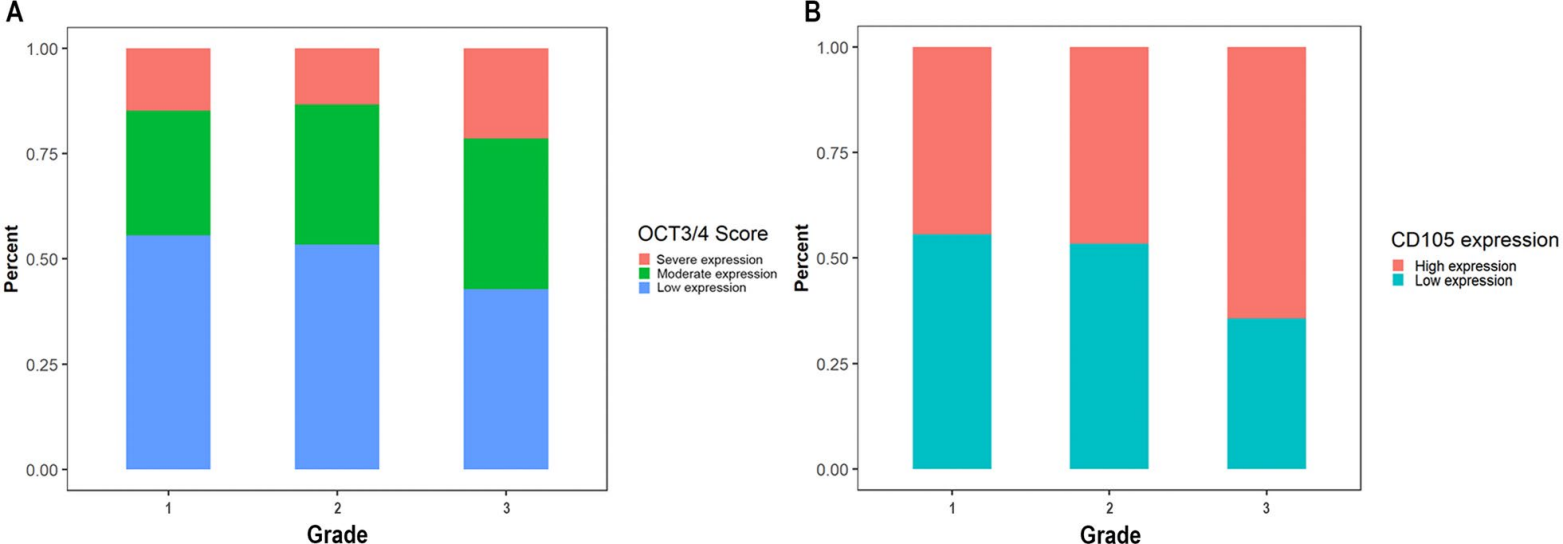
Antibodies expression in different tumor grades. **A** OCT3/4 expression in different tumor grades (*p* > 0.05). **B** CD105 expressions in different tumor grades (*p* > 0.05)

**Fig. 3:**
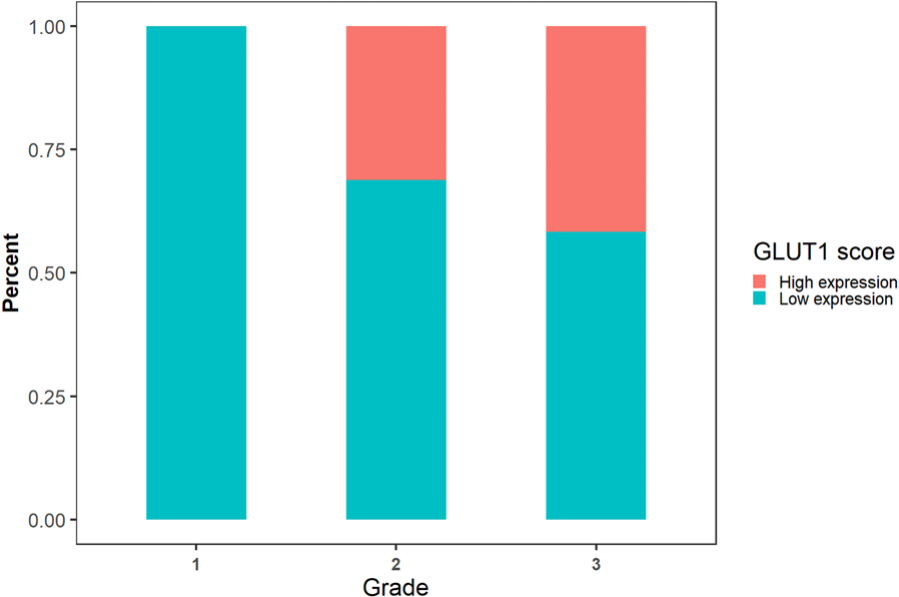
Expression of GLUT1 in different tumor grades (*p* = 0.002)

**Fig. 4:**
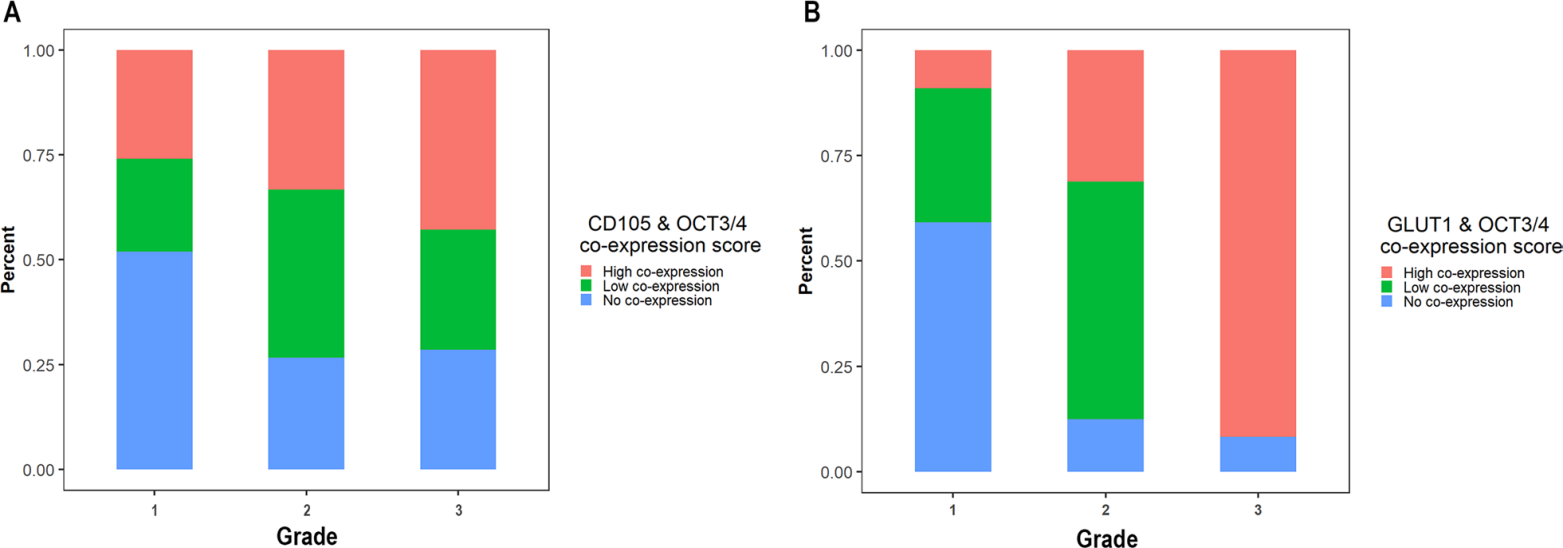
**A** Co-expression of CD105 and OCT3/4 in different tumor grades (*p* > 0.05). **B** Co-expression of GLUT1 and OCT3/4 in different tumor grades (*p* < 0.001)

Regarding the OCT3/4 expression final score, 29 samples scored as low expression, 18 samples scored as moderate expression, and nine samples scored as severe expression. There was not any significant correlation between tumor grade and expression of CD105 or OCT3/4 (Tables 3, 4 and Fig. 2). Six samples were excluded in step 2 for GLUT1 evaluation because of technical errors. The final score of GLUT1 revealed that all the samples with histological grade I had low expression of GLUT1. Using fisher’s exact test revealed statistically significant differences in GLUT1 expression among different tumor grades (*p* = 0.002) (Table 5 and Fig. 3). Evaluating the co-expression of antibodies revealed that there were no significant differences between the co-expression of CD105 and OCT3/4 in different grades of OSCC (Tables 3, 4 and Fig. 4). Regarding co-expression of GLUT1 and OCT3/4, 91.7% of the cases with histological grade III demonstrated high expression of antibodies. Fisher’s exact test revealed significant differences regarding co-expression of GLUT1 and OCT3/4 in different tumor grades (*p* < 0.001) (Table 6 and Fig. 4).

**Table 3:** Correlation of CD105 and OCT3/4 expression with tumor grade

	Grade	CD105	Co-expression*	OCT3/4
Grade				
Spearman correlation	1	0.145	0.244	0.095
Sig. (2-tailed)	–	0.28	0.07	0.484
N	56	56	56	56
CD105				
Spearman correlation	0.145	1	0.592	0.454
Sig. (2-tailed)	0.25	–	< 0.001	< 0.001
N	56	56	56	56
Co-expression*				
Spearman correlation	0.244	0.592	1	0.689
Sig. (2-tailed)	0.07	< 0.001	–	< 0.001
N	56	56	56	56
OCT3/4				
Spearman correlation	0.095	0.454	0.689	1
Sig. (2-tailed)	0.484	< 0.001	< 0.001	–
N	56	56	56	56

*Co-expression of both CD105 and OCT3/4 markers

**Table 4:** Expression of CD105 and OCT3/4 in different tumor grades

	CD105	Co-expression*	OCT3/4
Kruskal–Wallis H	1.515	3.319	0.682
*df*	2	2	2
Asymp. sig	0.46	0.19	0.71

*Co-expression of both CD105 and OCT3/4 markers

**Table 5:** Expression of GLUT1 in different tumor grades

		GLUT1 score		Total
		Low expression	High expression	
Grade				
I	Count	22	0	22
	% within grade	100.0%	0.0%	100.0%
II	Count	11	5	16
	% within grade	68.8%	31.3%	100.0%
III	Count	7	5	12
	% within grade	58.3%	41.7%	100.0%
Total	Count	40	10	50
	% within grade	37.5%	62.5%	100.0%

**Table 6:** Co-expression of GLUT1 and OCT3/4 in different tumor grades

		Co-expression score*			Total
		No co-expression	Low co-expression	High co-expression	
Grade					
I	Count	13	7	2	22
	% within grade	59.1%	31.8%	9.1%	100.0%
II	Count	2	9	5	16
	% within grade	12.5%	56.3%	31.3%	100.0%
III	Count	1	0	11	12
	% within grade	8.3%	0.0%	91.7%	100.0%
Total	Count	16	16	18	50
	% within grade	32.0%	32.0%	36.0%	100.0%

*Co-expression of both GLUT1 and OCT3/4 markers

As shown in Table 7, the histological grade was significantly correlated with GLUT1 scores and co-expression of GLUT1 and OCT3/4 (*p* < 0.05).

**Table 7:** Correlation of GLUT1 and OCT3/4 expression with tumor grade

	Grade	GLUT1	Co-expression*
Grade			
Spearman correlation	1	0.4461	0.6505
Sig. (2-tailed)	–	0.001	< 0.001
N	50	50	50
GLUT1			
Spearman correlation	0.4461	1	0.4632
Sig. (2-tailed)	0.001	–	< 0.001
N	50	50	50
Co-expression*			
Spearman correlation	0.6505	0.4632	1
Sig. (2-tailed)	< 0.001	< 0.001	–
N	50	50	50
OCT3/4			
Spearman correlation	0.6499	0.618	0.6974
Sig. (2-tailed)	< 0.001	< 0.001	< 0.001
N	50	50	50

*Co-expression of both GLUT1 and OCT3/4 markers

## Discussion

Recent studies support the correlation of CSCs and angiogenesis in different carcinomas. The population of CSCs increases in hypoxia, which results in rapid tumor growth and metastasis [19]. Also, CSCs can produce higher levels of vascular endothelial growth factor, one of the most important angiogenesis-inducing factors [20], than other malignant tumoral cells [21]. Thus, the combination of anti-angiogenetic drugs and anti-CSC drugs together with conventional cancer therapy is highly promising [22].

Studies on OSCC have shown the fundamental role of GLUT1 and CD105 in hypoxic response and the correlation of their expression with aggressive behavior and worse clinical outcomes [12, 17, 23]. Also, studies on OSCC demonstrated OCT3/4 as a potential prognostic marker correlated with poor overall survival rate and suggested further studies on its expression in conjunction with other markers [24].

Considering the overexpression of GLUT1 in pre-cancerous lesions, it may have a more prominent role in the early stages of malignant transformation [25], whereas CD105 represents the formed vessels and is associated with late stages of angiogenesis and hypoxic response. We used these two markers in our study to elucidate the role of CSCs in the early and late stages of the hypoxic response.

Our results suggest that in the early stage of the hypoxic situation in OSCC, CSCs may be responsible for hypoxic response due to overexpression of both GLUT1 and OCT3/4. However, in the late stage of the hypoxic response, which leads to angiogenesis and blood vessel formation, the expression of CSC markers is not significant, which means a reduction in the role of CSCs in angiogenesis in the late stage of the hypoxic situation.

Some recent studies showed tumor hypoxia was associated with a poorer prognosis in head and neck cancers [26, 27]. Our results regarding the significant correlation between co-expression of OCT3/4 and GLUT1 and tumor grade showed that CSCs could be associated with poorer prognosis via GLUT1 expression in hypoxic conditions. Further studies are mandatory to discover other accurate roles and associated pathways of CSCs in OSCC.

Recently, CSCs have been identified as potentially initiating cells of tumor angiogenesis and neovascularization. Evidence strongly suggests that the association of CSCs with tumor angiogenesis may be mediated through the induction of vascular endothelial growth factor production [28]. Also, CSCs can organize a pseudo-vascular network in some malignant tumors [28]. It might be the result of their differentiation into a borderline transitional cell type between an endothelial cell and a tumor cell among tumoral cells. Further studies are necessary to evaluate these processes. CSCs are diverse and have various markers. They vary in different grades and stages of a tumor, in different tumors, and also in different patients with the same type of tumor. We evaluated only one CSC marker (OCT3/4), which was a limitation of the present study. Another limitation of our study was the lack of TNM staging of the patients to evaluate the association of co-expression of antibodies with nodal or distant metastasis. It seems that assessment of the association between co-expression of antibodies and the prognosis of OSCC patients should be considered in further studies.

In recent years, the utilization of dual staining methods has considerably increased. Two or more antigens can be evaluated simultaneously by using dual staining. This method can reveal the correlation of different antigens and may also increase the chance of cross-reactions. Various procedures have been suggested to reduce these cross-reactions, such as using antibodies from different species [29].

To our knowledge, it is the first study to evaluate the expression of GLUT1 and CD105 as markers of hypoxia and angiogenesis in association with one CSC marker in OSCC via dual immunohistochemistry. We showed that CSCs could play an important role in the initial stages of the hypoxic situation and angiogenesis. Further studies are required to discover the roles, biomarkers, and associated pathways of CSCs in OSCC.

## Data Availability

The datasets used and/or analyzed during the current study available from the corresponding author on reasonable request.

## Abbreviations

OSCC: Oral squamous cell carcinoma
CSCs: Cancer stem cells
IHC: Immunohistochemistry.
OCT3/4: Octamer-binding transcription factor 3/4
GLUT1: Glucose transporter 1
CD105: Endoglin
